# The Impact of China’s ETS on Corporate Green Governance Based on the Perspective of Corporate ESG Performance

**DOI:** 10.3390/ijerph20032292

**Published:** 2023-01-27

**Authors:** Riquan Yao, Yingqun Fei, Zhong Wang, Xin Yao, Sasa Yang

**Affiliations:** 1 Huzhou Power Supply Company, State Grid Zhejiang Electric Power Co., Ltd., Huzhou 313000, China; 2 China Center for Energy Economics Research, School of Economics, Xiamen University, Xiamen 361005, China

**Keywords:** ETS, green governance, ESG, green innovation, government supervision

## Abstract

To achieve China’s “dual carbon” and common prosperity goals, corporate green governance is crucial. A key tool for promoting green growth is environmental legislation, particularly market-based regulation. With China’s carbon emission trading as a natural experiment, we adopt the DID method to quantitatively compare the gap between ESG performance of pilot and non-pilot carbon trading enterprises before and after policy implementation, thereby examining the impact, mechanism and optimization conditions of market-based environmental policy on corporate green governance based on panel data of China’s A-share listed companies from 2007 to 2019. In addition, PSM-DID and other methods are employed for preventing estimation bias caused by sample self-selection bias. It is found that: (1) the green governance level of pilot firms can be considerably improved by a carbon emission trading scheme (ETS); (2) the ETS primarily encourages enterprises to uphold their ESG obligations through increasing regulatory pressure from the government and corporate involvement in clean innovation; (3) enhancing regional marketization can strengthen the impact of carbon trading policy, and enterprises that are large and non-state-owned exhibit better performance with regard to green governance as a result of carbon trading policy. This paper provides practical experience for promoting corporate green governance to achieve the “dual carbon” goal based on a market mechanism from a micro perspective.

## 1. Introduction

The subjects of addressing environmental and climate issues have drawn attention of countries all over the world in recent years. To achieve high-quality development, China must overhaul its resource- and pollution-intensive production patterns. Reducing energy use and emissions has become crucial for addressing environmental issues. In 1992, China signed the United Nations Framework Convention on Climate Change, and the Paris Agreement requires parties to the Convention to make explicit national autonomous contributions to climate change mitigation to achieve a net zero increase in carbon emissions by mid-century and limit the global surface temperature increase to 2 °C relative to the pre-industrial revolution by the end of the century. Most countries have a clear timeline for carbon neutrality following achievement of peak carbon emissions. To address environmental pollution and demonstrate determination to combat global climate change, China committed to peak carbon by 2030 and to be carbon neutral by 2060 in 2020.

As a result, over the course of the “14th Five-year Plan”, encouraging transition to a green economy and fostering integration of the environment and economy have become crucial objectives of economic growth. Enterprises are the primary forces behind economic expansion but also the main sources of energy consumption and pollution emissions [1]. To address environmental issues, it is essential to alter corporate behavior, motivate companies to take on social and ecological responsibilities and adjust production processes to release emission reduction potential. This is critical for China to achieve dual objectives of balancing environmental governance and economic growth under the high-quality development pattern. Therefore, it has become urgently necessary to find a solution to the issue of how to encourage corporate production mode upgrades, thereby achieving green governance.

Given the negative effects of environmental pollution on society and individuals, enterprises may not have the initiative and enthusiasm to reduce emissions on their own, so government intervention is required. In response to climate change, China has made great efforts in emission reduction and energy conservation and implemented a series of mandatory administrative control measures. As claimed by the “Porter Hypothesis”, environmental regulation will compel firms to adopt clean technology and drive green innovation. This can help enterprises to obtain compensation effects through enhancing the quantity and quality of environmentally friendly products and the efficiency of their production procedures to cope with and make up for the high costs brought about by environmental regulation [2]. However, as the nature of public goods, environmental resources can easily lead to the phenomenon of “tragedy of the commons”, with enterprises transferring production to regions with lower regulatory intensity [3]. Increased environmental control has been demonstrated to be detrimental to economic expansion [4]. Compared to the lack of flexibility and the long-term incentive effect of mandatory administrative control, market-based environmental regulation policies use economic resource allocation to realize emission control and emission reduction of polluting enterprises and assign economic value to abatement behaviors [5], which is a more endogenous incentive [6]. As a typical market-based environmental policy, a carbon ETS makes enterprises trade emission quotas in the carbon market by granting carbon emission rights quotas. Firms with comparatively low costs for decreasing emissions can benefit from an ETS to trade by trading their remaining carbon emission permits [7]. This assists internalization of the benefits from emissions reductions, thus incentivizing enterprises to reduce their emissions. An additional attribution of an ETS is that it allows firms with high emission reduction costs to internalize environmental costs by purchasing emission licenses that exceed their own quotas from the market. This can force enterprises to invest in green technology, promoting development of clean technologies and helping to achieve development of a low-carbon and sustainable economy.

Green governance of enterprises includes three aspects: environment, society and governance (ESG). According to ESG performance, investors can objectively evaluate enterprises and choose whether to invest [8,9]. As a non-profit activity, green governance needs to be driven by external forces. The existing literature mainly focused on a single dimension, such as corporate social responsibility or environmental performance, and investigated how to improve these areas based on various perspectives, including social concerns [10,11], media pressure [12], civilized cities [13], green financial policies [14,15], corporate internal structure [16] and other viewpoints. The impacts of fulfilling corporate social responsibility on business operation are also explored [17,18].

Environmental regulation is a significant external aspect that must be taken into consideration. Meanwhile, there has been limited research on how environmental legislation affects green governance [19,20]. Some literature mainly discussed the role of regulation on regional pollution emissions and corporate innovation and confirmed the success of China’s ETS in facilitating development of green technology [21,22,23], promoting carbon efficiency [24], inhibiting carbon emissions [25,26,27] and the synergistic effect on pollution reduction and economic development [28,29]. These studies have provided macro and micro evidence for the efficacy of an ETS in China.

However, for corporate green governance behavior with sufficient externalities, few existing studies have focused on the impact of market-based environmental regulations. Corporate ESG reflects the specific scores of corporate environmental, social and corporate governance responsibilities, which can quantify the green governance performance of enterprises to some extent. Therefore, the main purpose of this paper is to explore the impact of China’s carbon ETS as a representative of market-based environmental regulation on corporate green governance based on the perspective of corporate ESG performance to answer the following questions: first, in the Chinese context, can the carbon trading system effectively motivate enterprises to fulfill their environmental and social responsibilities when focusing on their own governance and improve the overall green governance of enterprises? Second, what are the mechanisms through which implementation of an ETS changes corporate behavior? Third, what are the external and internal factors that affect the effectiveness of carbon trading implementation? Answering these questions can help to improve China’s carbon trading system and use the market to stimulate green governance of enterprises to unleash the potential of emissions reduction so as to achieve the goal of “double carbon” and sustainable development.

Based on the above, we examine how carbon trading policy affects corporate ESG performance using China’s A-share listed enterprises data from 2007 to 2019 and the DID method, aiming to gain insights into how market-based regulations impact corporate behaviors. These are the probable minor contributions of this paper: first, we examine, from the ESG standpoint, the effects of carbon trading policy on corporate green governance and enrich the empirical evidence on the role of environmental regulation based on the market with China’s ETS as a natural experiment; second, we explore the path through which carbon trading policies affect enterprise ESG performance and expand the channels through which carbon trading policies affect corporate behavior choices; third, we analyze how internal and external conditions affect ETS implementation, which, in some cases, can provide a reference for policymakers to enhance China’s ETS and create a national market for carbon emissions trading.

We organize the remaining sections of this paper as follows: we develop hypotheses based on the literature review in Section 2. In Section 3, we construct an econometric model, choose variables and explain the data source. In Section 4, we conduct an empirical analysis to assess the effects of an ETS on corporate ESG performance and confirm the effect based on robustness tests and examine the impact mechanisms. In Section 5, heterogeneous effects of China’s ETS on corporate ESG performance are tested. In Section 6, we provide the main conclusions and discuss the related policy implications.

## 2. Literature Review and Hypothesis Development

Most studies argue that market mechanisms play a more essential role in emission abatement and energy conservation than administrative supervision by the government [30,31,32,33]. An ETS is a major tool of environmental regulations that are based on market mechanisms. Based on the Coase property right theorem [34], it sets a carbon emission limit for enterprises by issuing carbon emission quotas and grants enterprises access to environmental resource property rights. With the help of a carbon price and carbon trading mechanism, entrusting the externality of the environment to the enterprise mainly produces an innovation compensation effect [35,36]. Specifically, by purchasing carbon emission quotas, the negative externalities of pollution are internalized into corporate costs [37,38], and, by selling carbon emission quotas, the positive externalities of pollution control are internalized into corporate benefits. For enterprises, when the cost of carbon is more expensive than the marginal cost of reducing emissions, they are more driven to adopt environment- and energy-friendly strategies and to fulfill environmental and social responsibilities in order to accumulate excess allowances that can be sold on the market for additional benefits [39]. For enterprises with high emissions and marginal emission reduction costs, under limited carbon allowances, in order not to bear an additional allowance cost, they must actively carry out independent innovation, optimize energy use structure, improve carbon efficiency and transition to cleaner production modes so as to alleviate the cost pressure of emission control [40] and achieve a long-term mechanism of sustainable development. Thus, corporate ESG performance can be improved. Therefore, the first hypothesis is proposed.

**Hypothesis** **1.** *The performance of corporate green governance can be efficiently improved by carbon emission trading policy.*

From the standpoint of government, the carbon emissions trade market is unique and emerging and has the characteristics of an environmental protection market, energy market and financial market. Carbon trading policy covers many firms that belong to monopoly industries and can shift the cost of purchasing quotas to the price of products, which can make huge profits [41,42]. Some enterprises engage in rent-seeking tactics to achieve policy goals [43,44], bringing about market failure for carbon trading. In order to optimize the mechanisms of carbon emissions trading in China, the government needs to carry out strict market supervision to prevent market abuse, price manipulation and market deception. Therefore, regulatory pressure on the government will also be reinforced upon installation and enhancement of the carbon trading system. What is more, some studies consider the government to be the main promoter of environmental protection and other social responsibility issues [45,46,47], and it is a crucial topic that motivates enterprises to undertake social obligations [48]. Therefore, corporate green governance capacity can be strengthened by a carbon trading scheme by increasing government supervision pressure.

From the perspective of enterprises, if they face cost constraints of carbon trading but keep the production technology and existing production methods unchanged, buying carbon emission allowances and reducing carbon emissions will lead to loss of profits and decreased market competitiveness. With the goal of maximization, enterprises will choose to develop green technology to emit less carbon in order to meet finite carbon emission quotas and benefit from trading excess quotas. Some findings in the literature suggest that China’s ETS is a valid tool in stimulating corporate green innovation [49,50], with no significant adverse effects on corporate production or employment [51]. On the one hand, technical innovation is the primary force for enhancing effectiveness of controlling and reducing environmental pollution [52] and increasing corporate social and environmental performance. Additionally, it can promote production efficiency of enterprises [53], thereby optimizing the governance performance of the enterprises. Therefore, carbon trading can enhance the performance of green governance by promoting corporate green technology. The previous analysis can be concluded as the second hypothesis.

**Hypothesis** **2.** *China’s ETS can stimulate enterprise green governance mainly by strengthening government regulatory pressure and promoting corporate green innovation.*

## 3. Methodology and Data

### 3.1. Econometric Models

#### 3.1.1. DID Model

We evaluate the role of the ETS on corporate green governance by constructing a DID model. DID is a standard method for assessing policy effect. It assumes that there is a difference between the change trends of the control and treatment groups before and after implementation of the policy on the premise that the policy only influences the treatment group while the control group is not affected. The DID method can measure the causal effect of the policy by capturing and quantifying this change gap. We apply the DID method to assess the effect of China’s ETS by observing the difference in change in green governance levels between pilot and non-pilot firms before and after the implementation of the policy to assess the specific impact of the ETS on corporate green governance.

As some developed economies, such as the European Union, have used carbon trading policy to promote carbon emissions and economic decoupling, China also introduced a carbon emission trading scheme by issuing the “Notice on Carrying out the Pilot Work of Carbon Emissions Trading” in 2011. As part of this initiative, seven pilot regions were started from 2013 to 2014 in Tianjin, Beijing, Shanghai, Hubei, Chongqing, Shenzhen and Guangdong. Eight high-energy-consuming industries, including electricity, heat production and supply, petrochemicals, chemicals, building materials, steel and paper, non-ferrous metals and aviation, are covered by the policy.

Therefore, the A-share listed enterprises from the eight industries in seven pilots are chosen as the treatment group and other listed companies as the control group. Companies that are marked with *ST, PT and ST and those with missing data are excluded. All companies included in the financial and insurance industries are removed. Since China identified the first batch of carbon trading pilot regions in 2011 but officially launched the carbon trading market in these regions around 2013, we choose 2013 as the time when the policy was implemented. For the purpose of identifying the relationship between carbon trading policy and enterprise ESG performance, we design the specific DID model as follows:

(1) $$ESG_{it}=\alpha_{0}+\alpha_{1}\left(treat_{it}\times post_{it}\right)+\beta control_{it}+\mu_{i}+industry_{j}\times year_{t}+\varepsilon_{it}$$

where *i*, *t* and *j* represent the enterprise, year and industry, respectively; *ESG* is the explained variable, representing the green governance performance of the enterprises; treat is the dummy variable of enterprise, and the value is 1 for pilot enterprises, 0 for non-pilot enterprises; post is the dummy variable of time, and its value is 0 for the years before 2013, 1 for 2013 and later years; control is the factor that may affect the green governance performance of the enterprise; $\mu_{i}$ is the fixed effect of the enterprise; $industry_{j}\times year_{t}$ is an industry–time fixed effect that captures the impact that time-variant and time-invariant industry characteristics may have on the estimated results; $\varepsilon_{it}$ is a random error term. $\alpha_{1}$ is the coefficient of the interaction term of interest, $treat_{it}\times post_{it}$. We can evaluate and judge how carbon trading policy affects corporate green governance through the magnitude and significance of $\alpha_{1}$. If $\alpha_{1}$ is significantly greater than 0, it indicates that the ETS can effectively motivate the green governance behavior of enterprises and vice versa, that the policy has no significant effect on corporate green governance performance.

#### 3.1.2. Intermediary Effects Model

This paper adopts a two-step model of mediation effect, and it is configured as follows so as to analyze the mechanism effects of the ETS on corporate green governance performance.

(2) $$M_{it}=\delta_{0}+\delta_{1}\left(treat_{it}\times post_{it}\right)+\varphi control_{it}+\mu_{i}+industry_{j}\times year_{t}+\varepsilon_{it}$$

(3) $$ESG_{it}=\tau_{0}+\tau_{1}M_{it}+\eta control_{it}+\rho_{t}+\mu_{i}+province_{j}\times year_{t}+\varepsilon_{it}$$

Among these, M represents the mediating variables, which respectively represent the pressure of government regulation and corporate green innovation. First, Equation (2) is constructed to evaluate the impact of the ETS on the two mediators, while Equation (3) is used to judge the effect of the mediators on corporate green governance. The mediation effect is evaluated using the significance of $\delta_{1}$ and $\tau_{1}$. If the coefficients of both are significantly non-zero, the mediating effect exists and the mechanisms can be further explained and claimed by the sign of the two coefficients.

### 3.2. Data and Variable Construction

Dependent variable: enterprise green governance performance (ESG), enterprise green governance is generally represented by environmental, social and corporate governance. We use the ESG information disclosure scores of sample enterprises published in the Bloomberg database to measure the dependent variable [54].

Control variables: (1) enterprise size (*size*), calculated as logarithm of enterprise’s annual total assets [21]; (2) net profit margin of total assets (*roa*), calculated as average balance of total assets divided by net profit margin [23]; (3) cash flow ratio (*cfr*), represented by proportion of cash flow, which is yielded from the business activities of the enterprise, to total assets [55]; (4) debt-to-asset ratio (*debt*), calculated by the ratio of enterprise’s liabilities to its total assets [56]; (5) ratio of independent directors (*ind*), expressed as the proportion of independent directors to the total number of board directors [54].

Mediating variables: (1) government regulatory pressure (*gov*), considering data limitations, the law and regulation scores in the marketization index of each region of China are used as proxy variables [57]; (2) enterprise green innovation (*lngpat*), representing enterprise’s attention on green development and the level of innovation, and can be measured by the logarithmic of the total number of corporate green patent applications [23].

The specific descriptions related to each variable are presented in Table 1.

In order to obtain balanced panel data, the time span of our study is from 2007 to 2019. The ESG performance data of enterprises are mainly from Bloomberg Environmental, Social, and Governance Database. Other variables at the enterprise level are mainly from the Wind and CSMAR databases, and the statistical yearbooks of China’s provinces are the primary data source at the regional level.

**Figure 1:**
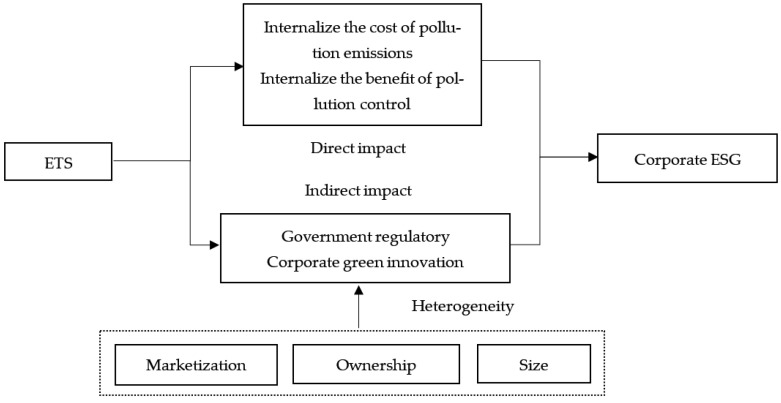
Procedure figure.

In this paper, we first apply the DID model to examine the specific effects of China’s ETS on the level of corporate green governance, then examine the impact mechanism based on the mediating effect model and finally examine the external and internal factors affecting the policy effect. The research design of this paper is shown in Figure 1.

## 4. Results

### 4.1. The DID Regression Results

To estimate Formula (1), we employ the DID method. Table 2 displays the results, where did stands for the cross-product term of the policy time dummy variables with the pilot enterprises dummy variable. The estimated effect of ETS on performance of corporate green governance without control variables is shown in column (1), and the average treatment effect after introducing control variables is shown in column (2). To further dynamically show the policy effect, column (3) introduces time trend items after the year when ETS was implemented. Further, *current* represents the policy impact on the green governance performance of enterprises in the implementation year, 2013, and post_N shows the effect of the Nth year after implementing the policy. All three models have controlled the firm fixed effects and industry–time fixed effects.

Column (1) and (2) show that the policy coefficients are both positive at the 5% significance level with and without introducing control variables. It can be concluded that China’s ETS is helpful to enhance corporate green governance, motivate enterprises to fulfill their environmental, social and corporate governance responsibilities and improve the performance of corporate green governance, which is meaningful for transformation of corporate green production. Therefore, Hypothesis 1 can be verified.

The results in Column (3) indicate that, in the start and first year of implementing the policy, corporate green governance is not significantly impacted by the carbon trading system. The coefficient during the second period of implementing the policy is considerably positive at the level of 10%. With time, the coefficient of the policy becomes more and more significant, and the degree of effect shows a trend of increasing annually. For the fourth year after implementing the policy, the incentive effect on corporate green governance reaches the maximum, indicating that the influence of the ETS on corporate green behavior exhibits a lagging characteristic. After China’s ETS was launched, enterprises needed time to adjust their existing production models.

### 4.2. Parallel Trend Test

**Figure 2:**
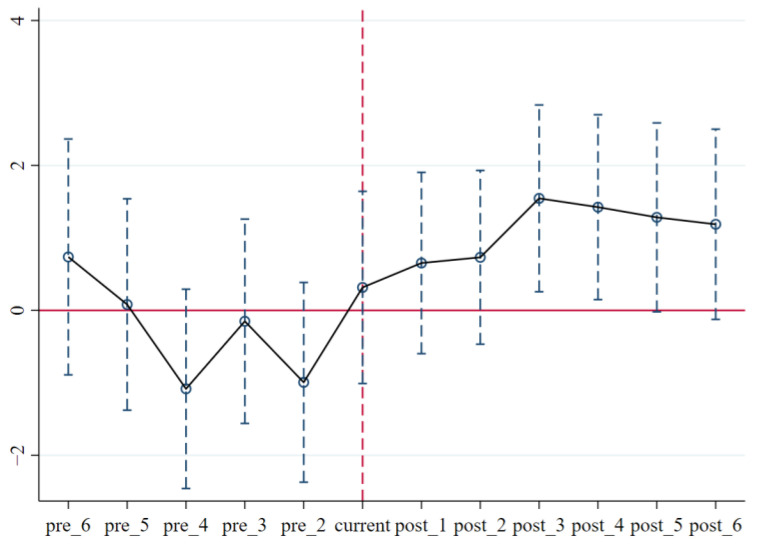
Parallel trend test.

The supposition of parallel trend, which states that there exists no discernible difference in the development trend of the performance of green governance between the control group and treatment group prior to when the ETS was implemented, must be satisfied in order for the DID method to be effective. For the purpose of assessing the parallel trend of the ETS, we introduce the time trend terms, pre_N, which is prior to the ETS’s implementation. The results of regression are presented in Column (4) of Table 2, and the corresponding parallel trend graph is shown in Figure 2.

The parallel trend assumption is met, as can be seen from the fact that the coefficients of time trend terms are not significant before the policy year, and the coefficient value fluctuates around 0, implying that no discernible difference between the pilot enterprises prior to and after the policy’s implementation exists. The validity and reliability of the results of the above analysis are suggested, implying that China’s ETS does exhibit a motivation effect on corporate green governance.

### 4.3. Robustness Checks

#### 4.3.1. PSM-DID Method

The method of PSM-DID is employed to conduct robustness tests, aiming to address the issue that the DID model is unable to ensure that the characteristics of enterprises in the control group are similar to those in the treated group prior to policy implementation and overcome estimation bias that is a result of the systematic differences between pilot enterprises and other enterprises. We use corporate control variables to identify the features of enterprises, based on which we can employ the PSM method to find and match similar enterprises of the control group to the treated group. DID regression is performed on the matching results, and column (1) of Table 3 presents the results. Disparities between the control and treated groups are obvious after introduction of the ETS based on the PSM-DID method. The regression outcome is similar to the previous one, supporting the above conclusion and demonstrating that the ETS is potent in enhancing corporate environmental, social and governance performance.

#### 4.3.2. Placebo Test

**Figure 3:**
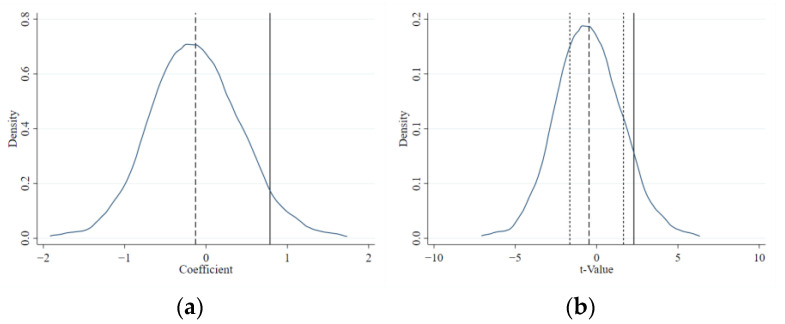
(**a**) Placebo test of coefficient; (**b**) placebo test of T-value.

Other unknowable factors may have an impact on the choice of experimental groups in examining the impact of ETS on the level of corporate green governance. A placebo test is necessary to confirm the validity of the conclusions reached in this research. For details, 500 samplings were carried out in the sample enterprises, the virtual experimental group was randomly selected, while other enterprises were used as the control group and regression was carried out again. The policy’s coefficient and distribution of kernel density about the T-value are illustrated in Figure 3a,b, respectively. The results show that the sampling estimation coefficient values are mainly distributed around 0, and the mean of the T-value corresponding to the sampling regression coefficient is within the range of the T-value corresponding to 10% significance. It demonstrates that the ETS shows no discernible effect according to the 500 random samplings, indicating that the aforementioned findings are the result of the policy and unrelated to other factors.

#### 4.3.3. Change the Time of Policy Implementation

Additionally, the carbon emissions trading policy can be tested to be effective if corporate green governance does not differ significantly over time without the policy based on artificially setting the policy’s implementation time. In this paper, we set 2010, 2011 and 2012 as the launch year of the ETS for regression, respectively. The fictional DID variables are named did2010, did2011 and did2012, respectively. Table 3’s column (2) to column (4) display the outcomes of the counterfactual test, which reveal that the coefficients of the three critical policy times cannot meet the significance level, indicating that, prior to 2013, the ETS had no discernible impact on the performance of green governance for the pilot enterprises. It shows that the improvement in the green governance performance of the pilot enterprises is indeed driven by the ETS, and it verifies the effectiveness of market-based regulatory policies. Accordingly, Hypothesis 1 is verified.

### 4.4. Mediation Effect Analysis

The findings above indicate that establishment of an ETS in China can facilitate corporate green governance. Then, the path through which this effect is achieved needs to be further tested. According to the theoretical hypothesis and based on the econometric models of Equations (2) and (3), from the views of regulatory pressure of government and green innovation of enterprises, we identify the mechanism effects of carbon trading policy on green governance of enterprises. Table 4 presents the findings. Columns (1) and (3) present the policy effects on government regulatory pressure and corporate green innovation, respectively. Columns (2) and (4) present the results of regression of government regulatory pressure and corporate green technology innovation on corporate ESG, respectively.

The outcomes of regression in Column (1) of Table 4 indicate that carbon trading policy shows positive effects on government regulatory pressure at the significance level of 1%, with a coefficient of 1.54. The results in Column (2) demonstrate that the coefficient of government regulatory pressure on corporate green governance performance is positive and meets the significance level of 1%. Combining the implication of Columns (1) and (2), implementation of China’s ETS will motivate enterprises to fulfill their ESG responsibilities by strengthening government regulatory pressure. The main reason is that China’s carbon trading market is in the early stage of construction. The marketization level of each region remains to be improved. The carbon emission trading policy relying on the market mechanism and related laws needs to be further improved. Additionally, most of the carbon trading pilot enterprises are monopolistic, which will trigger a series of market failures and violations of market order, such as rent-seeking and transferring environmental regulation costs to product prices. With implementation of carbon trading, to ensure efficacy and stable operation of the carbon trading market, higher requirements will be put forward for the government’s supervision. Government regulation strengthening can effectively compensate for carbon trading market failure and make enterprises pay more attention to improvement of their own green governance efficiency rather than improper pursuit of profits and avoidance of environmental regulation costs so that the carbon trading policy can achieve the effect of improving the green governance performance of enterprises.

The results in Column (3) show that the regression coefficient of China’s ETS on corporate green technology innovation is 0.20, which meets the 1% significance level, and the results in Column (4) indicate that corporate green technology innovation has a favorable impact on green governance performance, with a coefficient of 0.13. These suggestions illustrate that China’s ETS will stimulate enterprises to fulfill their green governance responsibilities by promoting their green technology innovation. The reason is that, from the firm’s perspective, carbon trading policy internalizes pollution emissions as costs of firms and pollution control as revenues of firms by allocating carbon emission shares and allowing the emission shares to be traded in the market. In order to maximize profits under environmental regulations, enterprises need to transform their production methods and technological structures to fundamentally reduce the amount of polluting output, and green technology innovation is an important means for enterprises to reduce pollution while improving production efficiency to “reduce costs and increase revenues”. What is more, green technology, as an important production technology, can help to improve corporate environmental performance and governance efficiency, thus realizing the green governance effect of carbon trading policy. In summary, Hypothesis 2 is validated.

## 5. Heterogeneity Analysis

### 5.1. Heterogeneity of Marketization Level

First, we test whether heterogeneity of regional marketization levels affects the green governance effect of carbon trading policy. Because carbon trading is reliant on market processes, its liquidity will have an impact on the cash flow generated by exchange of surplus allowances [58]. Market-oriented development of pilot areas will inevitably affect ETS efficacy. Based on the median level of regional marketization, we examine the heterogeneous policy effects on corporate green governance performance in two regions—those with a lower marketization level and those with a higher marketization degree. Columns (1) and (2) of Table 5 exhibit the results of the green governance effects of carbon trading policy in regions with high and low marketization, respectively.

It is evident that China’s ETS shows a favorable impact on performance of corporate green governance in regions where the marketization level is higher at the significance level of 10%, while the coefficient of the policy is negative for firms in regions with a lower degree of marketization, indicating that lack of marketization is unfavorable for the policy effect. The reason is that carbon trading relies upon market mechanisms and regions with lower marketization cannot effectively deal with a series of dysfunctions in the emerging carbon trading market, leading to environmental rent-seeking behavior of enterprises, weakening or even inhibiting the green governance effect of the ETS. This suggests that enhancing market mechanisms is conducive to giving full play to the incentive effects of China’s ETS on fulfilling social, environmental and governance responsibilities. As a result, when implementing environmental policies that are based on a market mechanism, it is important to consider the marketization process difference across various regions.

### 5.2. Heterogeneity of Enterprise Ownership

Second, we consider the impact of heterogeneity of enterprise ownership on carbon trading policy effect. The above analysis confirms that carbon trading policy can reinforce corporate green governance performance based on all A-share listed enterprises in China. However, enterprises with different ownership have different production models and social functions, and state-owned enterprises are pioneers in implementing government public policies [59] to fulfill social responsibility and respond to the national development strategy, which are the main goals instead of maximizing profits, and cost elasticity is small and may not respond strongly to environmental regulations. The carbon trading policy heterogeneity effect on different enterprises because of various ownership is partially concealed by the test using the whole listed companies sample. To investigate heterogeneous policy effects resulting from corporate ownership, we split the whole sample into two subsamples: state-owned and non-state-owned enterprises. Columns (3) and (4) of Table 5 reveal the outcomes of regression for enterprises with various ownership.

We can conclude that, for SOEs, the coefficient of the ETS is insignificant, while the policy exhibits favorable influence on the green governance of non-SOEs at the 1% level, showing the heterogeneity of the effect of carbon trading policy on corporate green governance with different ownership, probably due to the special nature of SOEs to fulfill their responsibilities and respond to national development strategies, and the costs of SOEs are less sensitive in the face of market-based environmental policies. What is more, SOEs are generally pillar enterprises of the national economy and have higher costs and longer cycles to reform their production models, so they cannot respond timely to environmental regulations in the short term. Non-SOEs, on the other hand, aim at profit maximization and are bound to take effective measures to adjust their production strategies and transition to green governance in the face of costs arising from environmental policies. Therefore, under a market-based environmental policy, enterprises that are non-state-owned are the main force for society to achieve emission reduction targets.

### 5.3. Heterogeneity of Enterprise Size

Then, we consider the green governance effects of the ETS for firms of different sizes. Carbon trading policy may have non-uniform effects on enterprises of different scales. Larger enterprises are more capable of fulfilling social responsibilities, and large firms have scale effects, better management systems and more efficient technology flows, so corporate teams can absorb the knowledge of their members with less effort; low knowledge acquisition costs are crucial for corporate innovation and production [60]. Therefore, considering the carbon trading market, strong economic and technological foundations of large enterprises can contribute to green innovation and scale emission reduction. We split the samples into small and large enterprises according to median annual operating revenue to shed light on how enterprise size influences efficacy of carbon trading policy. Table 5’s columns (5) and (6) display the outcomes of the two subsamples.

The findings demonstrate that, while the coefficient for small businesses is negligible, the coefficient for large businesses is highly positive, showing that the carbon trading scheme primarily raises the level of green governance at large enterprises. The main reason is that large enterprises have economies of scale, strong technological support and perfect internal governance systems and can quickly change their strategies and achieve green governance regarding an ETS. On the contrary, the assets and technology of small enterprises are insufficient to support their rapid response to environmental policies, and their green governance costs are higher, so carbon trading policy cannot have a significant policy effect on small enterprises. Therefore, large enterprises should take advantage of their economies of scale to take on environmental, social and governance responsibilities upon the launch of the national market for carbon trading and strive to shift to green and sustainable production.

## 6. Conclusions

Due to the seriousness of global climate change and recurrence of extreme weather, China, which leads the world in energy consumption and pollution emissions, as well as enterprises, which are the primary source of production and pollution, should change their strategy and behavior. Sparing no effort to encourage enterprises to enhance green governance and improve their performance in fulfilling environmental, social and governance responsibilities is crucial in achieving the “dual carbon” goal. Based on this, we employ a panel dataset of China’s A-share listed enterprises from 2007 to 2019 using ESG performance to measure corporate capacity for green governance to analyze the influence of an ETS on corporate green governance based on DID model construction. We also verify the validity and reliability of the results, explore the impact mechanisms and finally assess the external and internal conditions for strengthening the effects of China’s ETS. The main conclusions and contributions of the paper are as follows.

First, an ETS in China can considerably boost corporate green governance performance and, through a market mechanism, generate effective motivation for enterprises to fulfill their social and environmental responsibilities while enhancing their governance level. In addition, it is important to note that the effect of an ETS is lagging, showing that the policy effect gradually increases with time.

Second, China’s carbon emissions trading policy achieves the incentive effect on corporate green governance mainly by enhancing government supervision pressure and promoting green technology innovation of enterprises.

Third, in the case of China’s carbon trading policy implementation, green governance level of enterprises will be influenced by ownership characteristics and scale characteristics, and the regional marketization process is also an important prerequisite to determine whether the carbon trading policy is effective. China’s ETS mainly shows green governance effects on enterprises in regions with higher marketization levels, non-state enterprises and large enterprises but suppresses green governance levels of enterprises in regions with lower marketization levels and does not have significant green governance effects on state-owned enterprises and small enterprises.

According to the previous conclusions, policy implications can be suggested as follows: first, it is indispensable for the government to be conscious of the innovative compensation effect of environmental policies based on the market in the pursuit of the “dual carbon” goal. The government should take enterprises as the primary source of pollution control and the market as the policy orientation to realize internal management of environmental pollution costs and pollution control benefits based on the property rights system, and it can further provide stimulus for enterprises to uphold their social, environmental and governance obligations, hence fostering green and sustainable production and development. Second, effective implementation of a carbon trading policy is inseparable from government supervision. To maintain a functioning cycle and execution of the market where carbon emissions are continuously traded, the government should set strict and effective carbon trading laws and supervision systems. Additionally, government oversight should be fully utilized to promote corporate green governance and explore the potential for green transformation while offering solid assurance for accomplishing China’s “dual carbon” goal. Third, the government should actively encourage enterprises to innovate green technology and establish a sound system for protecting intellectual property to realize effective incentives for enterprise innovation and green transformation so that the carbon trading policy’s green innovation effect can be given full play and a technical foundation can be provided for enterprises to improve their social, environmental and governance capabilities. Fourth, when setting up the carbon trading mechanism, we should fully consider the characteristics of enterprises to maximize green governance advantages of non-state-owned and large enterprises and strengthen technology exchange among enterprises to stimulate enthusiasm of small firms and state-owned enterprises in green governance and encourage enterprises to transform to a greener model. The importance of market-oriented reforms should also be emphasized to ensure adequate market liquidity for carbon trading and strengthen the positive effects of the carbon market.

## Figures and Tables

**Table 1:** The description of variables.

Category	Symbol	Definition	Measurement
Dependent variable	*ESG*	Corporate green governance	The ESG information disclosure scores published in the Bloomberg database
Independent variable	*did*	Interaction terms for enterprise dummy variables and time dummy variables	$treat_{it}\times post_{it}$. treat=1 for treatment group; treat=0 for control group. post=1 for 2013–2019; post=0 for 2007–2012
Control variables	*size*	Enterprise size	The logarithm of the enterprise’s annual total assets
	*Roa*	Net profit margin of total assets	The average balance of total assets divided by net profit margin
	*cfr*	Cash flow ratio	The proportion of cash flow to total assets
	*debt*	Debt-to-asset ratio	The ratio of enterprise’s liabilities to its total assets
	*ind*	Ratio of independent directors	The proportion of independent directors to the total number of board directors
Mediating variables	*gov*	Government regulatory pressure	the law and regulation scores in the marketization index of each region
	*lngpat*	corporate green innovation	the logarithmic of the total number of corporate green patent applications

**Table 2:** Benchmark regression results.

	(1)	(2)	(3)	(4)
did	0.7869 **	0.7672 **		
	(0.3439)	(0.3478)		
pre_6				0.7373
				(0.8301)
pre_5				0.0808
				(0.7442)
pre_4				−1.0829
				(0.7015)
pre_3				−0.1506
				(0.7191)
pre_2				−0.9930
				(0.7030)
current			0.1137	0.3166
			(0.6162)	(0.6763)
post_1			0.7569	0.6536
			(0.5737)	(0.6382)
post_2			1.0133 *	0.7319
			(0.5493)	(0.6117)
post_3			1.6580 ***	1.5456 **
			(0.5983)	(0.6574)
post_4			2.0451 ***	1.4244 **
			(0.5819)	(0.6508)
post_5			1.4324 **	1.2835 *
			(0.6093)	(0.6648)
post_6			1.7618 ***	1.1880 *
			(0.6045)	(0.6695)
size		1.1050 ***	0.7977 ***	0.8679 ***
		(0.0884)	(0.0903)	(0.0900)
roa		0.7505	1.1458	0.9268
		(0.7787)	(0.7715)	(0.7715)
cfr		0.3865	0.2225	0.4782
		(0.6018)	(0.5967)	(0.5951)
debt		−1.7160 ***	−1.4934 ***	−1.2795 ***
		(0.4239)	(0.4192)	(0.4219)
ind		0.0068	0.0003	0.0016
		(0.0100)	(0.0099)	(0.0099)
cons	18.7788 ***	−5.9263 ***	1.1957	−0.5457
	(0.0356)	(1.9938)	(2.0410)	(2.0311)
Firm fixed effects	Yes	Yes	Yes	Yes
Industry–year fixed effects	Yes	Yes	Yes	Yes
Observations	10365	10365	10365	10365

Note: *, **, *** Significant at 10%, 5% and 1% confidence levels, respectively (the same below).

**Table 3:** Robustness tests.

		(1)	(2)	(3)	(4)
		psm_did	2012	2011	2010
did		0.8899 ***			
		(0.3412)			
did2012			0.3203		
			(0.3520)		
did2011				0.2632	
				(0.3723)	
did2010					0.3329
					(0.3921)
size		0.7424 ***	0.6925 ***	0.7804 ***	0.6697 ***
		(0.0917)	(0.0928)	(0.0906)	(0.0921)
roa		1.8324 **	1.4355 *	1.4028 *	1.5402 **
		(0.8057)	(0.7745)	(0.7706)	(0.7699)
cfr		0.0465	0.1990	0.0264	0.1174
		(0.6061)	(0.5948)	(0.5965)	(0.5965)
debt		−1.1016 ***	−1.0271 **	−1.1873 ***	−1.1211 ***
		(0.4221)	(0.4212)	(0.4202)	(0.4199)
ind		0.0004	0.0015	0.0003	−0.0002
		(0.0098)	(0.0098)	(0.0098)	(0.0098)
cons		2.2529	3.3453	1.4731	3.9722 *
		(2.0718)	(2.0934)	(2.0453)	(2.0792)
Firm fixed effects		Yes	Yes	Yes	Yes
Industry–year fixed effects		Yes	Yes	Yes	Yes
Observations		10355	10365	10365	10365

**Table 4:** Mediation effect tests.

	(1)	(2)	(3)	(4)
	gov	ESG	lngpat	ESG
did	1.5396 ***		0.2020 ***	
	(0.1401)		(0.0513)	
gov		0.0622 ***		
		(0.0207)		
lngpat				0.1344 **
				(0.0684)
size	−0.0459	0.8569 ***	0.2065 ***	0.7497 ***
	(0.0378)	(0.0904)	(0.0103)	(0.0912)
roa	0.6323 **	0.8561	−0.2443 **	0.9239
	(0.3196)	(0.7725)	(0.1153)	(0.7682)
cfr	−0.0543	0.2326	0.0034	0.2720
	(0.2486)	(0.5971)	(0.0918)	(0.5955)
debt	0.3535 **	−1.2845 ***	−0.2549 ***	−1.1523 ***
	(0.1729)	(0.4212)	(0.0632)	(0.4211)
ind	−0.0089 **	0.0042	−0.0012	−0.0003
	(0.0041)	(0.0099)	(0.0015)	(0.0098)
cons	10.6695 ***	−0.9382	−4.0355 ***	2.1330
	(0.8513)	(2.0171)	(0.2338)	(2.0579)
Firm fixed effects	Yes	Yes	Yes	Yes
Industry–year fixed effects	Yes	Yes	Yes	Yes
Observations	10365	10365	10365	10365

**Table 5:** Heterogeneity tests.

	(1)	(2)	(3)	(4)	(5)	(6)
	Marketization Level		Enterprise Ownership		Enterprise Size	
	high	low	soe	non-soe	big	small
did	0.8094 *	−1.6585 **	0.5397	2.1930 ***	1.2755 ***	−0.3100
	(0.4372)	(0.7540)	(0.4319)	(0.6280)	(0.4894)	(0.5238)
size	0.6954 ***	1.2101 ***	0.5663 ***	1.7630 ***	1.1652 ***	1.0988 ***
	(0.1416)	(0.1292)	(0.1532)	(0.1188)	(0.1826)	(0.1241)
roa	1.9021 *	−0.0617	1.3172	−1.2592	2.7506 *	−0.3038
	(1.1344)	(1.0847)	(1.3436)	(0.9517)	(1.6642)	(0.7928)
cfr	−1.2497	1.7774 **	0.6611	1.3995 *	−0.0863	−0.5368
	(0.8106)	(0.8929)	(0.8564)	(0.8497)	(0.9591)	(0.7252)
debt	−1.8063 ***	−1.4583 **	−1.6139 **	−2.0788 ***	−2.5452 ***	−0.3440
	(0.6207)	(0.5972)	(0.6281)	(0.6186)	(0.8170)	(0.4774)
ind	0.0389 ***	−0.0243*	0.0067	−0.0079	−0.0119	0.0187
	(0.0147)	(0.0135)	(0.0128)	(0.0167)	(0.0154)	(0.0124)
cons	2.4183	−7.3950 **	6.9526 **	−20.5661 ***	−5.5448	−7.3596 ***
	(3.1939)	(2.9247)	(3.4870)	(2.6857)	(4.2616)	(2.7309)
Firm fixed effects	Yes	Yes	Yes	Yes	Yes	Yes
Industry–year fixed effects	Yes	Yes	Yes	Yes	Yes	Yes
Observations	5069	5244	5416	4930	5144	5134

## Data Availability

Not applicable.
